# Enhanced electrical conductivity at Fe_3_O_4_ grain boundaries

**DOI:** 10.1126/sciadv.aeb8164

**Published:** 2026-05-01

**Authors:** Tingting Yao, Chunyang Gao, Ziyi Sun, Ang Tao, Yixiao Jiang, Zhiqing Yang, Xiu-Liang Ma, Hengqiang Ye, Chunlin Chen

**Affiliations:** ^1^ Shenyang National Laboratory for Materials Science, Institute of Metal Research, Chinese Academy of Sciences, Shenyang 110016, China.; ^2^ School of Materials Science and Engineering, University of Science and Technology of China, Shenyang 110016, China.; ^3^ Jiangxi Copper Technology Research Institute Co. Ltd., Nanchang 330096, China.; ^4^ Ji Hua Laboratory, Foshan 528200, China.; ^5^ Bay Area Center for Electron Microscopy, Songshan Lake Materials Laboratory, Dongguan 523808, China.; ^6^ Institute of Physics, Chinese Academy of Sciences, Beijing 100190, China.

## Abstract

Clarifying how grain boundaries (GBs) in materials affect the electrical property is critical to the design and application of electronic nanodevices. A common physical scenario is that GBs have lower electrical conductivity than bulk materials due to intense electrons scattering. In this work, we demonstrate that Σ5 and Σ13 GBs in Fe_3_O_4_ bicrystal thin films exhibit substantially enhanced electrical conductivity compared to the grain interior based on nano- to macroscale electrical measurements. The atomic and electronic structures of the GBs have been systematically investigated by combining aberration-corrected scanning transmission electron microscopy and first-principles calculations. It has been revealed that the enhanced electrical conductivity at the Fe_3_O_4_ GBs arises from a half-metallic–to–metallic transition, which is attributed to the spin-up conduction channel provided by tetrahedrally coordinated Fe sublattice. This study reveals the atomistic mechanism of GB-enhanced conductivity, thereby deepening the understanding of GB electrical property.

## Teaser

Fe_3_O_4_ grain boundaries–enhanced conductivity is attributed to a half-metallic–to–metallic transition.

## INTRODUCTION

The grain boundaries (GBs) of materials have garnered extensive and sustained attention owing to their critical role in modulating mechanical and physical properties (*1*–*5*). For structural materials, the strength usually increases with decreasing grain size due to the block of GBs to the dislocation motion, which is summarized as the well-known Hall-Petch relationship (*6*, *7*). For functional materials, GBs are often the birthplace of intriguing physical and chemical properties due to their unique atomic and electronic structures (*8*–*12*). To date, GB engineering has become an effective strategy to improve material performance and design new materials (*13*–*17*).

As for the influence of GBs on electrical properties, GBs typically exhibit lower electrical conductivity compared to the grain interior in most cases, primarily because of enhanced electron scattering and current blocking caused by defects (*18*–*21*). Certain high-angle tilt GBs in copper even display significantly larger resistivity than their low-angle tilt conterparts, because of high dislocation densities and the associated strain field (*22*, *23*). As for polycrystalline oxides such as Nb-doped SrTiO_3_, Y-doped BaZrO_3_, and TiO_2_, the GB blocking effect are attributed to the presence of space charge depletion layers (*24*–*27*). The generally low electrical conductivity of GBs often hinders the performance of related materials in electronic application. However, carrier transport in materials occurs not only across the GB but also through them, therefore, exploring feasible strategies to enhance GB electrical conductivity and elucidating that the underlying atomistic mechanisms are of important scientific value and practical significance in the field of electronic materials and devices.

Magnetite (i.e., Fe_3_O_4_) is a typical half-metallic compound with cubic inverse spinel structure, in which the tetrahedral sites (Fe_A_) are occupied by Fe^3+^ ions and the octahedral sites (Fe_B_) by equal amounts of Fe^2+^ and Fe^3+^ ions. The magnetic coupling between Fe_A_ and Fe_B_ ions are antiferromagnetic, which makes Fe_3_O_4_ a ferrimagnet with a net magnetic moment of 4 μB per formula unit (*28*). At room temperature, the arrangement of Fe^2+^ and Fe^3+^ ions in the B sites are random, and the rapid electron hopping between Fe^2+^ and Fe^3+^ ions contributes a high electrical conductivity of ~250 Ω^−1^·cm^−1^ (*29*). The band structure of spin-up electrons in Fe_3_O_4_ presents the characteristics of insulation, while that of spin-down electrons is metallic. Only the spin-down electrons contribute to the electrical conductivity of Fe_3_O_4_, which renders it to be half-metallic. From the view point of band structure, it is possible to enhance the electrical conductivity of GBs in Fe_3_O_4_ by changing the band structure of spin-up electrons from insulating to metallic.

In this study, Fe_3_O_4_ Σ5 and Σ13 GBs are fabricated through the epitaxial growth of bicrystal thin films on SrTiO_3_ bicrystal substrates using pulsed laser deposition (PLD). Macroscopic electrical performance measurements and conductive atomic force microscopy (AFM) reveal that the Fe_3_O_4_ GBs exhibit substantially enhanced electrical conductivity compared to the grain interior. A combined investigation using aberration-corrected scanning transmission electron microscopy (STEM) and first-principles calculations is conducted to elucidate the atomic and electronic structures of the GBs and to uncover the atomistic origin of the enhanced GB electrical conductivity.

## RESULTS

**Fig. 1.**
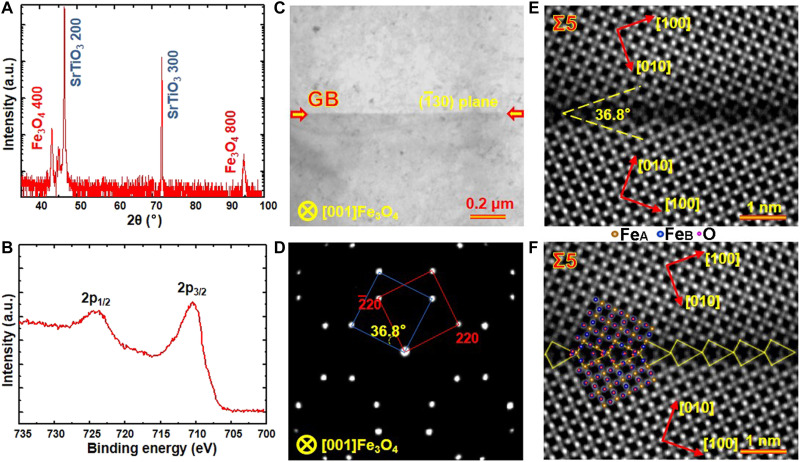
Structure characterizations of the Fe_3_O_4_ Σ5 bicrystal thin film. (**A**) Out-of-plane HRXRD pattern of the as-prepared Fe_3_O_4_ bicrystal thin film deposited on the SrTiO_3_ (001) substrate with a Σ5 GB. (**B**) XPS spectrum of the as-prepared Fe_3_O_4_ thin film. As-prepared Fe_3_O_4_ bicrystal thin film is composed of pure Fe_3_O_4_ phase and has a good crystallinity. (**C**) Bright-field TEM image and (**D**) SAED pattern of the Fe_3_O_4_ bicrystal thin film along the [001] zone axis. The GB is marked by the red arrows. (**E** and **F**) HAADF images of the Fe_3_O_4_ Σ5 GB with different labels. Schematic models are inserted in (F) to show clearly the atomic structure and structural units of the GB. a.u., arbitrary units.

Figure S1 schematically illustrates the epitaxial growth of an Fe_3_O_4_ bicrystal thin film containing a symmetric tilt GB on the SrTiO_3_ bicrystal substrate. The PLD process for depositing Fe_3_O_4_ epitaxial thin films has been described detailedly in previous research (*30*). By using SrTiO_3_ bicrystals with symmetric tilt angles of 36.8° and 22.6°, the Fe_3_O_4_ bicrystal thin films featuring Σ5 and Σ13 GBs are successfully fabricated, respectively. Figure 1A presents the high-resolution x-ray diffraction (HRXRD) pattern of the Fe_3_O_4_ Σ5 bicrystal thin film deposited on the SrTiO_3_ bicrystal substrate with a 36.8° tilt angle. The diffraction peaks can be indexed as the (400) and (800) diffraction peaks of Fe_3_O_4_ and the (200) and (400) diffraction peaks of SrTiO_3_. No additional periodic diffraction peaks are detected in the HRXRD pattern, indicating that the Fe_3_O_4_ thin film grows epitaxially on the SrTiO_3_ substrate and is composed of pure Fe_3_O_4_ phase. Figure 1B displays the Fe 2p x-ray photoelectron spectroscopy (XPS) spectrum obtained from the surface of the Fe_3_O_4_ thin film. The Fe 2p peak clearly splits into the Fe 2p_3/2_ (at ~710.3 eV) and Fe 2p_1/2_ (at ~724.3 eV) peaks, with no satellite peaks appearing between them. These features are consistent with the typical Fe 2p XPS spectrum of Fe_3_O_4_ (*31*–*33*). In contrast, the Fe 2p XPS spectrum of Fe_2_O_3_ and FeO exhibit a satellite peak located at approximately 718.8 and 715.5 eV between Fe 2p_3/2_ and Fe 2p_1/2_ peaks (*31*).

To directly characterize the atomic structure of the GB, TEM and STEM analyses were performed. Figure 1 (C and D) presents a representative plan-view bright-field TEM image and the corresponding selected area electron diffraction (SAED) pattern acquired from the Fe_3_O_4_ Σ5 bicrystal thin film along the [001] zone axis. The SAED pattern clearly reveals two sets of diffraction spots (marked with red and blue lines) separated by a tilt angle of 36.8°, indicating that the GB formed between the two adjacent grains is a Σ5 [001] ($\bar{1}$30) GB. This GB lies on the ($\bar{1}$30) crystal plane. Figure 1 (E and F) presents the atom-resolved high-angle annular dark-field (HAADF) STEM images of the Fe_3_O_4_ Σ5 GB, with different labeling schemes. As indicated in Fig. 1E, the measured tilt angle between two grains is 36.8°. An atomic structural model is inserted in Fig. 1F to provide a clearer illustration of the GB structural units.

**Fig. 2.**
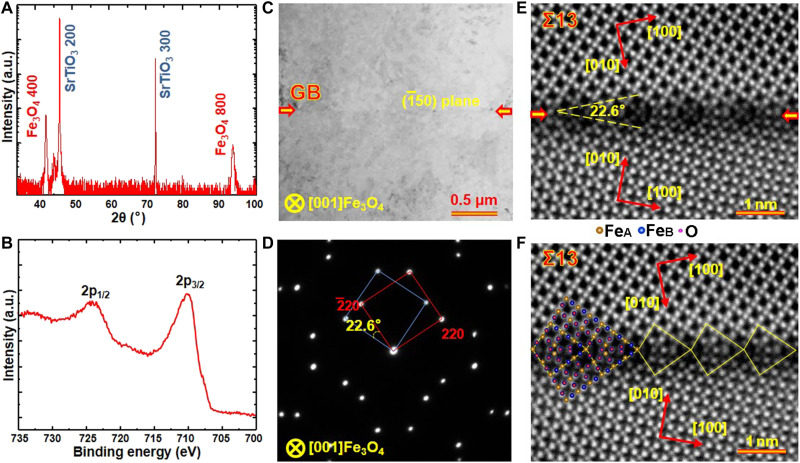
Structure characterizations of the Fe_3_O_4_ Σ13 bicrystal thin film. (**A**) Out-of-plane HRXRD pattern of the as-prepared Fe_3_O_4_ bicrystal thin film deposited on the SrTiO_3_ (001) substrate with a Σ13 GB. (**B**) XPS spectrum of the as-prepared Fe_3_O_4_ thin film. (**C**) Bright-field TEM image and (**D**) SAED pattern of the Fe_3_O_4_ bicrystal thin film along the [001] zone axis. The GB is marked by the red arrows. (**E** and **F**) HAADF images of the Fe_3_O_4_ Σ13 GB with different labels. Schematic models are inserted in (F) to show clearly the atomic structure and structural units of the GB.

Figure 2 shows microstructural characterization results of the as-prepared Fe_3_O_4_ Σ13 bicrystal thin film deposited on the SrTiO_3_ bicrystal substrate with a symmetric tilt angle of 22.6°. Similar to the results in Fig. 1, the HRXRD pattern and XPS spectrum in Fig. 2 (A and B) confirm that Fe_3_O_4_ Σ13 bicrystal thin film grows epitaxially on the SrTiO_3_ substrate and consists of a pure Fe_3_O_4_ phase with excellent stoichiometry. Figure 2 (C and D) shows the plan-view bright-field TEM image and the corresponding SAED pattern of the Fe_3_O_4_ Σ13 GB viewed along the [001] zone axis. The GB (indicated with arrows) is well-bonded without any secondary phase. According to the SAED pattern, the tilt angle between the adjoining grains is determined to be 22.6^o^ and the Σ13 GB is formed on the ($\bar{1}$50) crystal plane. Figure 2 (E and F) displays the atom-resolved HAADF STEM images of the Fe_3_O_4_ Σ13 [001] ($\bar{1}$50) GB, which clearly reveal the atomic arrangement within the GB structural units.

**Fig. 3.**
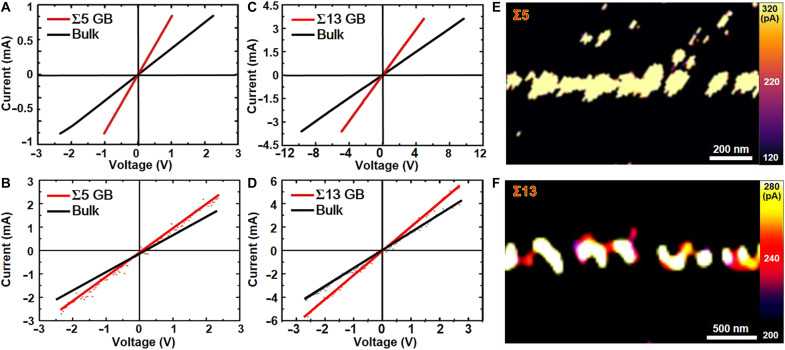
Electrical conductivity characterizations of the Fe_3_O_4_ bicrystal thin films. (**A** and **B**) *I-V* curves of the Fe_3_O_4_ Σ5 GB and the grain interior measured by two-point and four-point methods, respectively. (**C** and **D**) *I-V* curves of the Σ13 GB and the grain interior measured by two-point and four-point methods, respectively. (**E** and **F**) Typical current-mapping images near the Σ5 and Σ13 GBs obtained with the contact-current mode of AFM.

To investigate the electrical properties of Fe_3_O_4_ GBs, the room-temperature electrical conductivity of Σ5 and Σ13 GBs are measured using the two-point method with a Hall-effect measurement system and the four-point method with a SourceMeter measurement system. For comparison, the electrical conductivity of the bulk regions are also evaluated. The schematic diagrams depicting the experimental details of conductivity measurements are provided in fig. S2 (A and B). Figure 3 (A and B) displays the current-voltage (*I*-*V*) characteristics of the Fe_3_O_4_ Σ5 GB as measured by the Hall-effect system and SourceMeter system, respectively. As can be seen, the slopes of the *I-V* curve for the Σ5 GB (in red) are steeper than those for the Fe_3_O_4_ bulk region (in black), indicating higher electrical conductivity of the Σ5 GB compared to the bulk material. According to the quantitative data from the four-point measurement, the electrical resistance of the Σ5 GB is reduced by approximately 32.7% relative to that of the grain interior. The *I-V* characteristics of the Σ13 GB (in red) and the bulk region (in black) measured using the two-point and four-point methods are shown in the Fig. 3 (C and D, respectively). Consistent with the result for the Σ5 GB, the slopes of the red curves are substantially greater than those of the black curves, suggesting that the electrical conductivity of the Σ13 GB is also superior to that of the Fe_3_O_4_ bulk. On the basis of the statistical analysis of the four-point test date, the electrical resistance of the Σ13 GB is reduced by approximately 34.4% compared to the grain interior. To visually demonstrate the enhanced electrical conductivity at the Σ5 and Σ13 GBs, electrical conductivity measurements are carried out using the contact-current mode of conductive-probe AFM. Representative current-mapping images near the Σ5 and Σ13 GBs are shown in Fig. 3 (E and F, respectively). It is clear that both the Σ5 and Σ13 GBs exhibit higher electrical conductivity than the Fe_3_O_4_ bulk, which is in excellent agreement with the *I-V* characteristics.

**Fig. 4.**
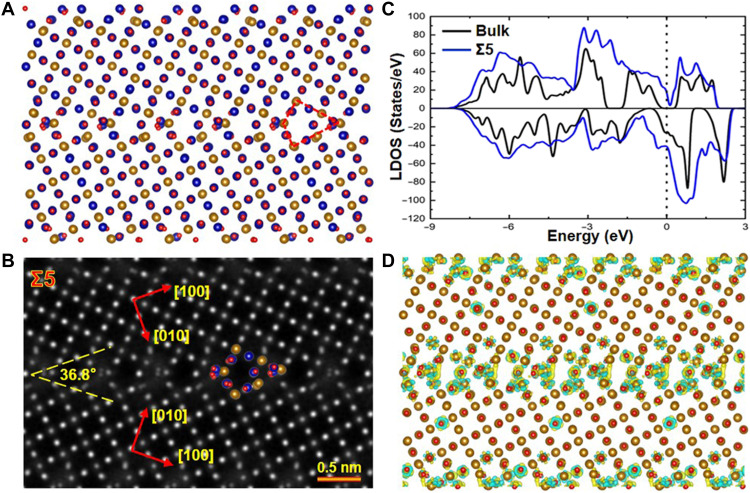
Atomic and electronic structure of the Σ5 GB obtained by first-principles calculations. (**A**) Atomic model of the Fe_3_O_4_ Σ5 GB. (**B**) Corresponding simulated HAADF image. (**C**) Spin-polarized LDOS of the Σ5 GB (blue line) and the bulk Fe_3_O_4_ (black line). (**D**) Charge difference density near the GB. Electrons are rich at the Σ5 GB, thereby resulting in an enhanced GB electrical conductivity.

First-principles calculations are carried out to investigate the origin of enhanced electrical conductivity at GBs. Figure 4A presents the atomic model of the Fe_3_O_4_ Σ5 GB obtained by the structural optimization. To determine the magnetic coupling across the GB, the interface energies of the Σ5 GB with ferromagnetic and antiferromagnetic couplings are calculated. The results suggest that the Σ5 GB prefers ferromagnetic coupling with an interface energy of 0.21 J/m^2^, while the antiferromagnetic coupling configuration exhibits a substantially higher interface energy of 0.56 J/m^2^.

The spin-polarized local density of states (LDOS) across the Σ5 GB are presented in fig. S3 (A to D). Both spin-up and spin-down states coexist at the Fermi level in the Σ5 GB. The preservation of spin polarization direction across the boundary confirms the ferromagnetic coupling between adjacent grains at the Σ5 GB. Figure 4B is the simulated HAADF image based on the atomic model in Fig. 4A. The simulated HAADF image matches well with the experimental one (i.e., Fig. 1E), indicating that the Σ5 GB atomic model is reasonable. The LDOS for both the Σ5 GB and bulk Fe_3_O_4_ are calculated and shown in Fig. 4C with the Fermi level marked by dotted lines. The blue curve represents the LDOS at the Σ5 GB region, and the black curve corresponds to the bulk Fe_3_O_4_. Notably, the Fermi level at the Σ5 GB is occupied by both spin-up and spin-down electrons, in contrast to the bulk Fe_3_O_4_, where only spin-down electrons are present at the Fermi level. This observation suggests that a half-metallic–to–metallic transition occurs at the Σ5 GB. The charge density difference across the Σ5 GB is illustrated in Fig. 4D, clearly showing electron accumulation at the Σ5 GB compared to the grain interior, which contributes to the enhanced electrical conductivity at the GB.

**Fig. 5.**
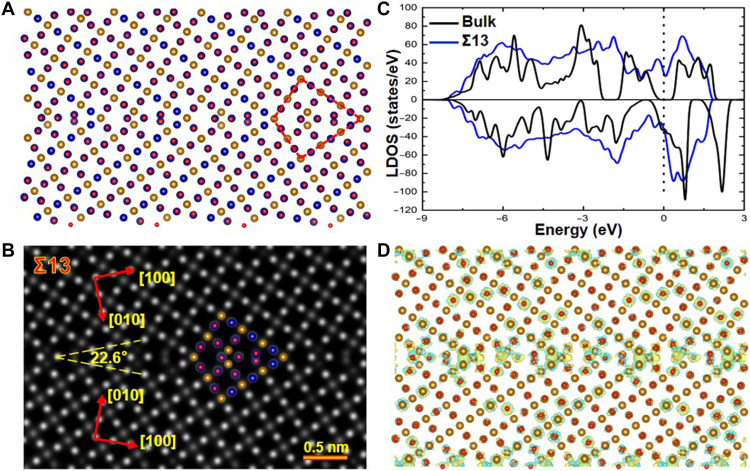
Atomic and electronic structure of the Σ13 GB obtained by first-principles calculations. (**A**) Atomic model of the Fe_3_O_4_ Σ13 GB. (**B**) Corresponding simulated HAADF image. (**C**) Spin-polarized LDOS of the Σ13 GB (blue line) and the bulk Fe_3_O_4_ (black line). (**D**) Charge difference density near the GB. Electrons are rich at the Σ13 GB, thereby resulting in an enhanced GB electrical conductivity.

Figure 5A shows the atomic model of the Fe_3_O_4_ Σ13 GB obtained by the structural optimization. Calculations reveal that the Σ13 GB tends to form the antiferromagnetic coupling with an interface energy of 0.58 J/m^2^, while the ferromagnetic coupling has an interface energy of 0.64 J/m^2^. The spin-polarized LDOS across the Σ13 GB are displayed in fig. S3 (E to H). Both spin-up and spin-down states are present at the Fermi level in the Σ13 GB, while the spin polarization reverses direction across the Σ13 GB, revealing its antiferromagnetic coupling character. Using this GB atomic model, we simulate the HAADF image of Σ13 GB and show the simulated image in Fig. 5B. The simulated HAADF image is well consistent with the experimental one (i.e., Fig. 2E), thereby confirming that the Σ13 GB atomic model is reasonable. The calculated LDOS of the Σ13 GB and the bulk Fe_3_O_4_ are shown in Fig. 5C with the Fermi level marked by the dotted line. The blue and black curves represents the LDOS of the Σ13 GB and the bulk Fe_3_O_4_, respectively. Similar to the case of Σ5 GB, the Fermi level of the Σ13 GB is also occupied by both spin-up and spin-down electrons, indicating that the Σ13 GB is also metallic because of the half-metallic–to–metallic transition. The charge density difference across the Σ13 GB is shown in Fig. 5D. The charge density at the GB is higher than in the grain interior, which leads to the enhanced GB electrical conductivity.

Since electrons accumulate at the Σ5 and Σ13 GBs, the valence states of Fe ions at these GBs will reduce compared to those in the bulk. To experimentally validate these predictions, the valence states of Fe ions were analyzed using electron energy loss spectroscopy (EELS) via a second derivative method (*34*, *35*). Figure S4 (A and B) presents representative EELS spectra of Fe-L_3_, Fe-L_2_, and O-K edges acquired from the Σ5 GB and the grain interior, respectively. No obvious energy shift was detected in these spectra. Figure S4C shows the corresponding second derivative spectra of Fe-L_3_ and Fe-L_2_ edges. By measuring the positive portions of the L_3_ and L_2_ peaks in the second derivative of the spectra (shaded regions), the L_3_/L_2_ ratios at the Σ5 GB and the grain interior are determined to be approximately 4.85 and 5.08, respectively. The smaller L_3_/L_2_ ratio at the Σ5 GB indicates a reduction in the valence states of Fe ions at the GB relative to the grain interior. Similar situations occur at the Σ13 GB. As shown in fig. S5, the L_3_/L_2_ ratios calculated from the positive peak areas in the second derivative spectra (shaded regions) are approximately 4.97 at the Σ13 GB and 5.12 in the grain interior, further confirming that the valence states of Fe ions at the Σ13 GB are lower than in the surrounding grains. These EELS results collectively demonstrate that the GBs are enriched with electrons, leading to a reduction in the valence state of Fe from Fe^3+^ to Fe^2+^, which is consistent with the calculated carrier accumulation at the interface.

## DISCUSSION

As shown in Fig. 4 (A and D), charge carriers accumulate simultaneously at both the Fe_A_ and Fe_B_ sites of the Σ5 GB. The charge accumulation at the Fe_A_ sites is the primary origin of the metallic transition in the system. Electrons occupy the initially unoccupied spin-up states of Fe_A_, thereby opening a spin-up conduction channel within this sublattice. This channel cooperates with the intrinsic spin-down conduction channel of the Fe_B_ sublattice at the GB, leading to pronounced metallic character in the interfacial region and a significant enhancement in interfacial conductivity. Similar to the Σ5 GB, charge accumulation also occurs at the Fe_A_ sites in the Σ13 GB, thereby enhancing the interfacial conductivity.

In summary, revealing the atomistic origin of GB conductivity is crucial for both fundamental physics and practical applications of electronic materials and devices. This study reveals that Σ5 and Σ13 GBs in Fe_3_O_4_ exhibit substantially enhanced electrical conductivity compared to the grain interior—a phenomenon that contrasts with the conventional view that GBs typically impede conductivity because of strong electron scattering. The electrons enrichment at the interface reduces the Fe valence state from Fe^3+^ to Fe^2+^. The tetrahedrally coordinated Fe sublattice provides a spin-up conduction channel, inducing a metallic transition at the interface and thereby enhancing the GB’s conductivity. This mechanism represents a type of transition from half-metallic to metallic state. This study elucidates the atomistic mechanism underlying GB-enhanced electrical conductivity, thereby providing insights for the understanding of the physical properties and potential functional applications of GBs. This strategy of enhancing GB electrical conductivity through half-metal–to–metal transition may be applicable to a broad range of half-metallic materials.

## MATERIALS AND METHODS

### Preparation of samples

The Fe_3_O_4_ bicrystal thin films with Σ GBs used in this study were deposited on SrTiO_3_ bicrystal substrates by PLD with an Fe_3_O_4_ target. The substrates were cleaned by ultrasonic washing in acetone and annealed at 600°C for 20 min to obtain a flat surface before deposition. For PLD deposition, the substrate temperature, oxygen pressure, energy of KrF laser (λ = 248 nm), and repetition rate were adopted as 700°C, 5 Pa, 1.5 J/cm^2^, and 5 Hz, respectively. After deposition, the Fe_3_O_4_ bicrystal thin films were cooled down to room temperature at a cooling rate of ~10°C/min. Using these parameters, Fe_3_O_4_ films can be grown with a unique orientation relationship with SrTiO_3_ substrate. During the epitaxial deposition of Fe_3_O_4_ films on these SrTiO_3_ bicrystal substrates, it was observed that the deposited Fe_3_O_4_ on both sides of the GB preserved the symmetric tilt geometry inherent to the substrate, thereby enabling the formation of Σ5 and Σ13 GBs. The TEM and STEM samples preparation procedures includes cutting, grinding, dimpling, and Ar ion-milling. The voltage and incident angle during Ar ion-milling were set from 4.5 to 0.3 keV and 8° to 4°.

### Structural characterizations and electrical conductivity measurements

Bright-field TEM images and the corresponding SAED patterns were recorded on a Tecnai F20 (FEI) TEM. HAADF STEM images and EELS spectra were obtained by using a 300-kV microscope (Titan Cubed Themis G2300, FEI). For HAADF imaging, a probe size of ∼1 Å, a probe convergence angle of ∼25 mrad, and collection semiangles of detectors of 68 to 280 mrad were adopted. The energy resolution of EELS spectra was 0.4 eV. HAADF images were simulated using Dr. Probe package developed by J. Barthel, which is based on the multislice method (*36*). The simulations parameters were applied on the basis of the parameters of STEM experiments, for instance, the probe convergence angle as ~25 mrad, collection semiangle as 68 to 200 mrad, and Cs as 0.02 mm. The EELS spectra were collected with a dispersion of 0.15 eV per channel. This dispersion allows us to collect at the same time the Fe-L and O-K edges for the higher-energy edges.

The electrical conductivity of the GBs were analyzed by two-point and four-point methods and current-mapping image, respectively. The two-point method was carried out by Hall effect measurement system (K2500-3RSLP, MMR, USA). To reduce the contact resistance, indium was used as an electrode of the GBs to connect the probes of the Hall effect measurement system. The same test was carried out far away from the GBs as reference. The schematic diagram for experimental setting was shown in fig. S2A. To confirm ohmic contacting during the electrical test, *I-V* curves at GB and the bulk were measured with SourceMeter measurement system (SMU 2650, Keithley, USA). Four indium electrodes were made, and the distance between each electrode was 1 mm. The schematic diagram for experimental setting was shown in fig. S2B. During the electrical conductivity measurement with four-point method, a total of 100 date points were collected and subsequently fitted into an *I-V* curve. The current-mapping images were obtained by the AFM (Cypher, Asylum Research, UK) under a 2-V applied voltage using the contact mode with a Au-coated probe. The schematic diagram illustrating the experimental setup is presented in fig. S2C. Upon application of voltage, the conductive probe makes contact with the surface of Fe_3_O_4_ thin film. Subsequently, current flows from the probe into the film, then through the film to the sample stage, and finally back to the instrument, thereby forming a complete electrical circuit. By detecting the current passing through the sample, the spatial distribution of current flow within the sample can be obtained. The tip radius of the probe was 20 ± 10 nm. During the measurement, the Ohmic contact between Fe_3_O_4_ films and AFM bottom electrode was realized by conductive silver paint.

### DFT calculations

First-principles calculations based on density functional theory (DFT) (*37*, *38*) were carried out using the Vienna ab initio simulation package (*39*, *40*). A generalized gradient approximation in the form of the Perdew-Burke-Ernzerhof (*41*) generalized function was used. Projector augmented wave (*42*, *43*) pseudopotential method was used, where the plane wave energy cutoff was 400 eV. The Fe 3d^6^4s^2^ and O 2s^2^2p^4^ were treated as valence states. The structural optimization was performed until the Hellmann-Feynman forces were less than 0.03 eV/Å. For the bulk primitive cell of Fe_3_O_4_, a 7 × 7 × 7 Monkhorst-Pack k-point grid was used. The two interface-structured supercells used for the calculations contained 536 and 440 atoms, respectively. A full relaxation approach was used for the supercells. The interface energy of the GB was calculated by the following equation

$$E_{f}=\frac{E_{b}-E_{c}}{2A}$$

where *E*_*b*_ denotes the total energy of the GB model, *E*_*c*_ denotes the energy of the corresponding bulk with the same numbers of Fe and O atoms, and *A* is the area of the GB in the supercell.

Charge difference density was used to visualize the charge transfer by bonding across the interface using the following equation

ρ=ρ(AB)−ρ(A)−ρ(B)

where ρ(*AB*) is the interfacial model charge density and ρ(*A*) and ρ(*B*) are the charge densities of the structures on both sides of the interface, respectively.
