# Supplementary material for: Enhanced electrical conductivity at Fe3O4 grain boundaries
Source: Sci Adv. 2026 May 1;12(18):eaeb8164. doi: 10.1126/sciadv.aeb8164 (PMC13134580; doi:10.1126/sciadv.aeb8164)
Supplement: Supplementary file 1 — Figs. S1 to S5 [file sciadv.aeb8164_sm.pdf]

Science Advances
AAAS

Supplementary Materials for

Enhanced electrical conductivity at Fe_3O_4 grain boundaries

Tingting Yao *et al.*

Corresponding author: Chunlin Chen, clchen@imr.ac.cn

Sci. Adv. **12**, eaeb8164 (2026)
DOI: 10.1126/sciadv.aeb8164

This PDF file includes:

Figs. S1 to S5

Supplementary Figure 1

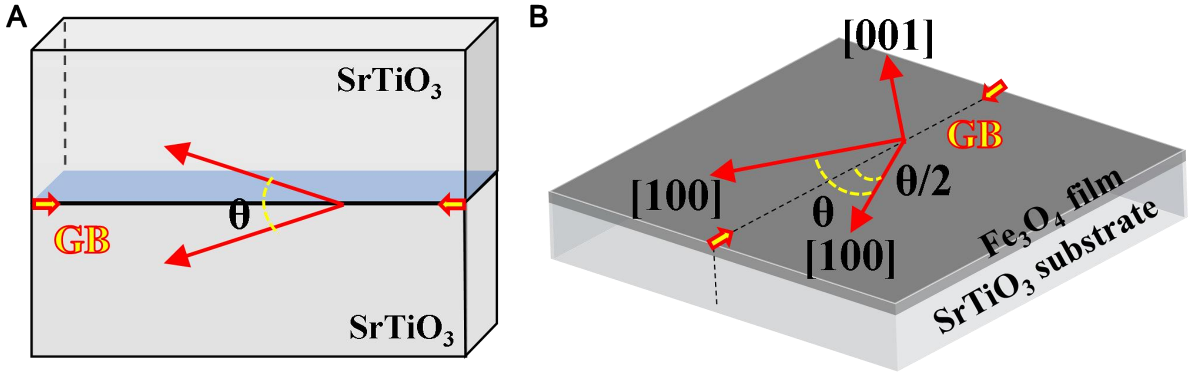

Fig. S1| (**A**) Schematic diagram of the orientation relationship of the $SrTiO_3$ bi-crystal substrate. (**B**) Schematic diagram of the preparation of Fe_3O_4 bi-crystal thin film with a single GB on the $SrTiO_3$ bi-crystal substrate.

Supplementary Figure 2

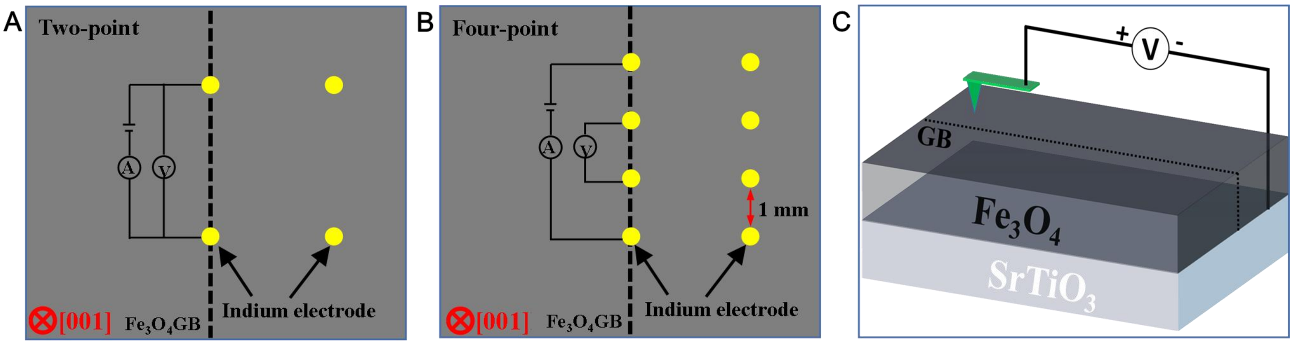

Fig. S2| Schematic diagrams for conductivity measurement. (**A**) Schematic diagram for the measurement of I-V characteristic curves by two-point method. (**B**) Schematic diagram for the measurement of I-V characteristic curves by four-point method. The GBs are denoted by dotted line, and the indium electrodes are marked by yellow dots. I-V characteristic curves at the GBs and the grain interior are measured. (**C**) Schematic diagram showing the measurement of current map near the GB using the contact-current mode of AFM. The dotted line represents the GB.

Supplementary Figure 3

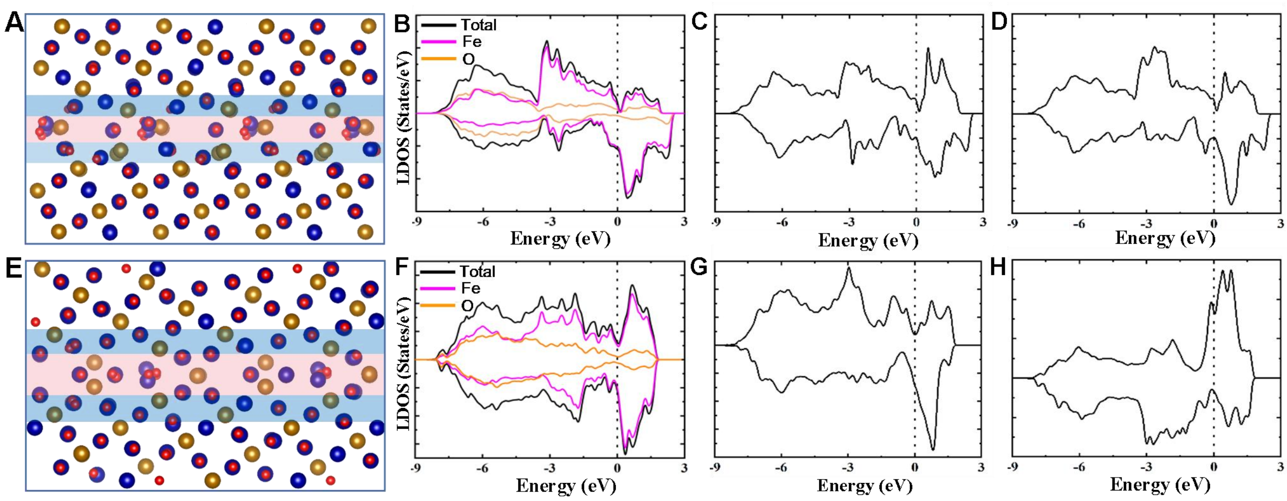

Fig. S3| Calculated LDOS across the Σ5 and Σ13 GBs. (**A**) Atomic model of the Fe_3O_4 Σ5 GB. (**B**) Spin-polarized LDOS of the Σ5 GB interface (pink area). (**C**) and (**D**) Spin-polarized LDOS for the regions near the Σ5 GB interface on both sides (blue area). (**E**) Atomic model of the Fe_3O_4 Σ13 GB. (**F**) Spin-polarized LDOS of the Σ13 GB interface (pink area). (**G**) and (**H**) Spin-polarized LDOS for the regions near the Σ13 GB interface on both sides (blue area). The spin polarization retains the same direction across the Σ5 grain boundary, revealing the ferromagnetic coupling nature. On the contrary, the Σ13 grain boundary tends to form the antiferromagnetic coupling.

Supplementary Figure 4

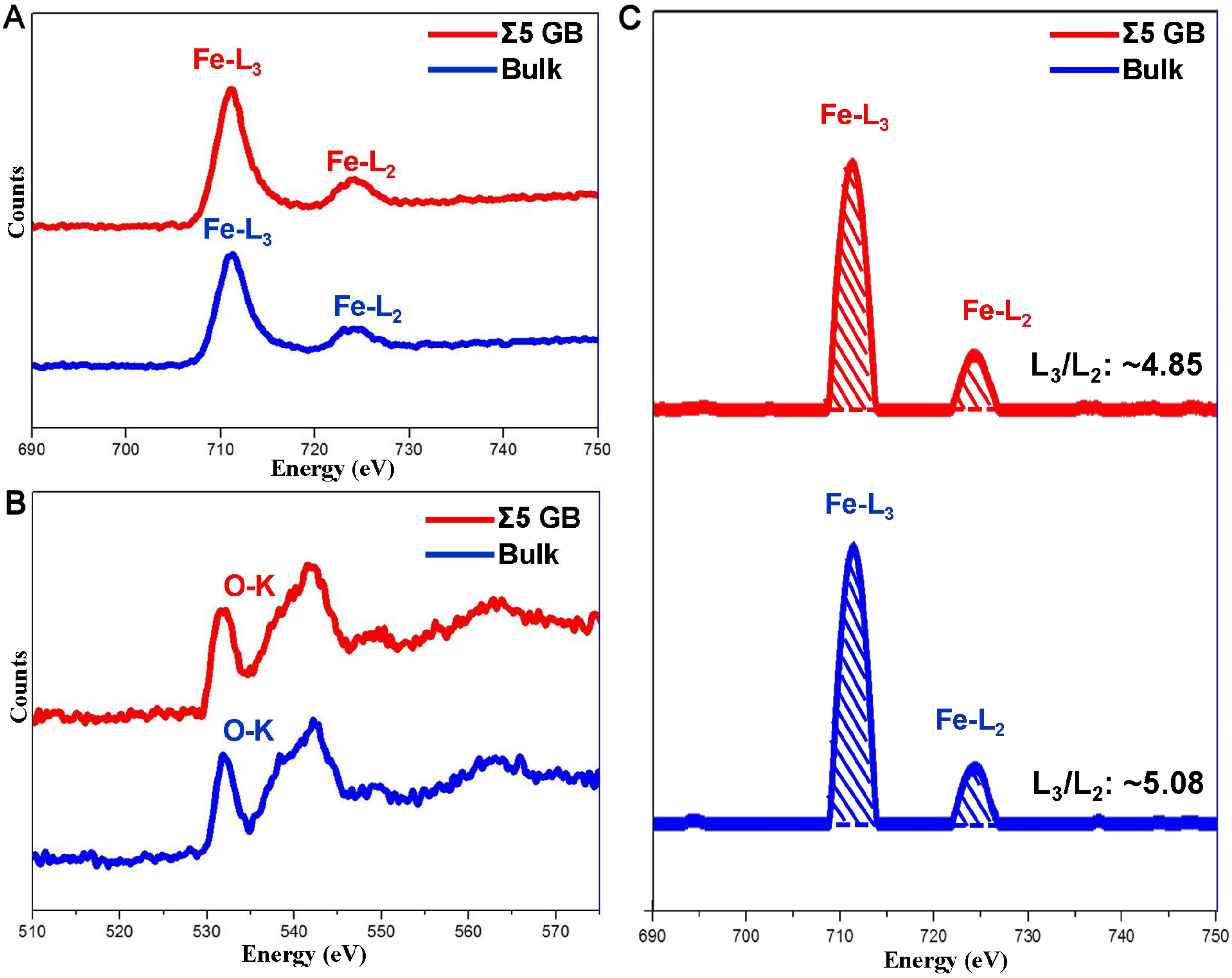

Fig. S4| EELS spectra showing the Fe-L and O-K edges obtained at the Fe_3O_4 Σ5 GB (red) and the grain interior (blue). (**A**) Fe-$L_{2,3}$ and (**B**) O-K edges. (**C**) The corresponding second derivative Fe-L edge spectra. The L_3/L_2 ratio is calculated by measuring the positive part of L_3 and L_2 peaks in shadow. The L_3/L_2 ratios at the Σ5 GB and the grain interior can be calculated as ~ 4.85 and ~ 5.08, respectively.

Supplementary Figure 5

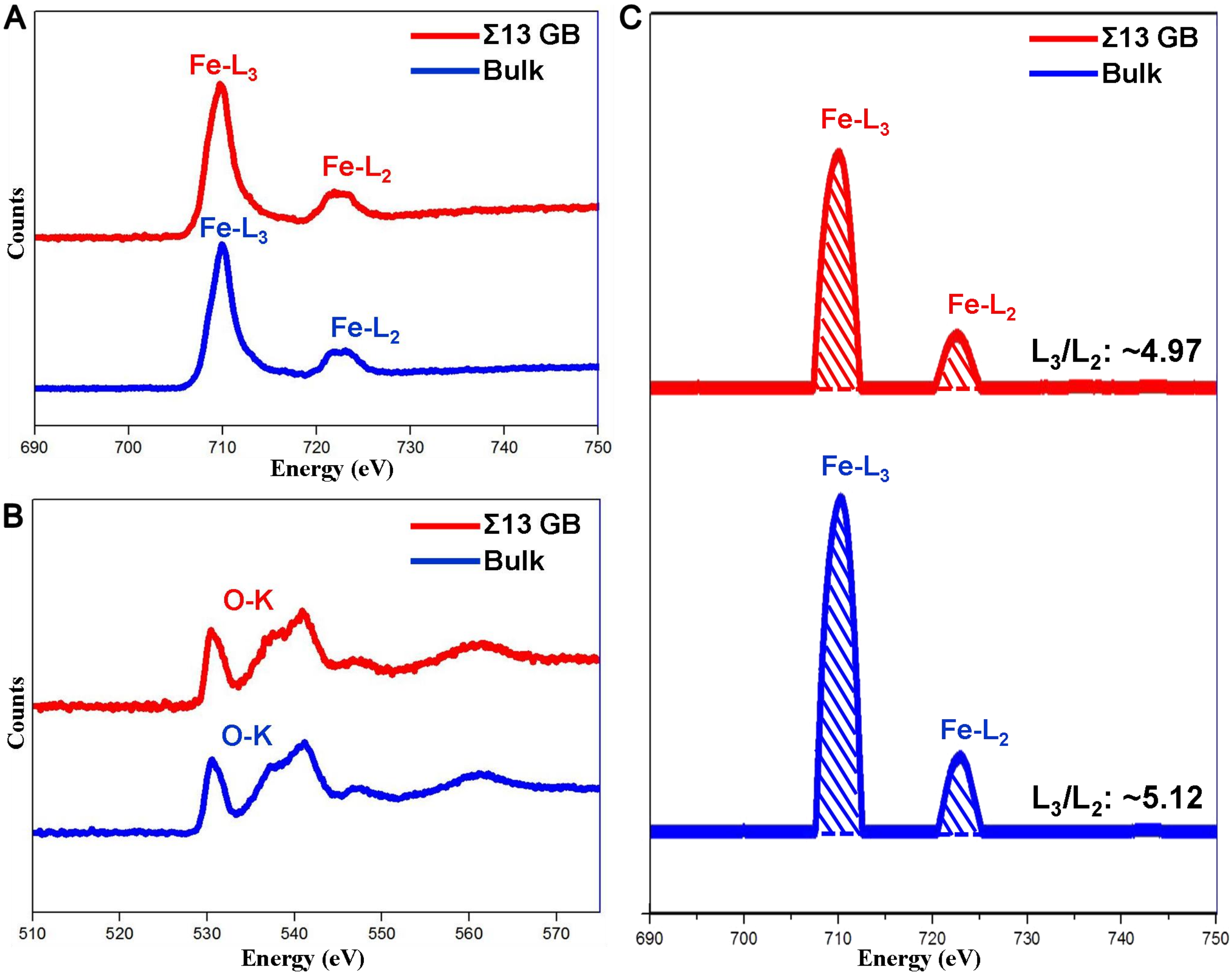

Fig. S5| EELS spectra showing the Fe-L and O-K edges obtained at the Fe_3O_4 Σ13 GB (red) and the grain interior (blue). (**A**) Fe-$L_{2,3}$ and (**B**) O-K edges. (**C**) The corresponding second derivative Fe-L edge spectra. The L_3/L_2 ratios at the Σ13 GB and the grain interior are calculated as ~ 4.97 and ~ 5.12, respectively.
